# A new interpretation of the bee fossil *Melitta willardi* Cockerell (Hymenoptera, Melittidae) based on geometric morphometrics of the wing

**DOI:** 10.3897/zookeys.389.7076

**Published:** 2014-03-14

**Authors:** Alexandre Dewulf, Thibaut De Meulemeester, Manuel Dehon, Michael S. Engel, Denis Michez

**Affiliations:** 1 University of Mons, Research Institute of Biosciences, Laboratory of Zoology, Place du parc 20, 7000 Mons, Belgium; 2 Naturalis Biodiversity Center, Darwinweg 2, PoBox 9517, 2300RA Leiden, the Netherlands; 3 Division of Entomology (Paleoentomology), Natural History Museum, and Department of Ecology and Evolutionary Biology, 1501 Crestline Drive – Suite 140, University of Kansas, Lawrence, KS 66045, U.S.A.

**Keywords:** Bees, compression, Oligocene, wing shape, geometric morphometrics, Tertiary

## Abstract

Although bees are one of the major lineages of pollinators and are today quite diverse, few well-preserved fossils are available from which to establish the tempo of their diversification/extinction since the Early Cretaceous. Here we present a reassessment of the taxonomic affinities of *Melitta willardi* Cockerell 1909, preserved as a compression fossil from the Florissant shales of Colorado, USA. Based on geometric morphometric wing shape analyses *M. willardi* cannot be confidently assigned to the genus *Melitta* Kirby (Anthophila, Melittidae). Instead, the species exhibits phenotypic affinity with the subfamily Andreninae (Anthophila, Andrenidae), but does not appear to belong to any of the known genera therein. Accordingly, we describe a new genus, *Andrenopteryx* **gen. n.**, based on wing shape as well as additional morphological features and to accommodate *M. willardi*. The new combination *Andrenopteryx willardi* (Cockerell) is established.

## Introduction

Bees (Hymenoptera, Apoidea, Anthophila) are a monophyletic group of largely pollenivorous species derived from among the predatory apoid wasps (Engel 2001a, 2011, Michener 2007). This clade probably appeared in the Early Cretaceous (~120 Myr) (Engel 2001a), and concomitant with the diversification of the Eudicots (Michener 1979, Cardinal and Danforth 2013). While intensive work during the last 20 years has clarified many aspects of bee relationships (e.g., Engel 2011, Danforth et al. 2013), establishing the tempo of this radiation continues to be hampered by significant gaps in their fossil record. Hitherto only 191 fossil species of bees have been described (Michez et al. 2012, Engel et al. 2012, Wappler et al. 2012, Engel and Breitkreuz 2013, Engel and Michener 2013, Engel et al. 2013), but the majority of these come from a relatively restricted number of actual deposits. Four main deposits of bee fossils are known: (i) the Eckfeld/Messel shales (47-44 Myr; Wappler and Engel 2003, Wedmann et al. 2009), (ii) the Baltic amber from the middle Eocene (45 Myr; Engel 2001a, 2008, Gonzalez and Engel 2011), (iii) the Florissant shale from the Eocene-Oligocene boundary (34 Myr; Zeuner and Manning 1976, Engel 2001b, 2002, unpubl. data) and (iv) the Dominican amber from the Miocene (20 Myr; Engel et al. 2012, Engel and Breitkreuz 2013). Specimens in amber are typically preserved with enough fidelity to correctly explore diagnostic morphological characters while compressions generally show a restricted subset of such features (Michener 2000, Engel 2001a). Taxonomic attributions of many compression fossils need objective and robust revision with modern procedures, such as geometric morphometrics (Michez et al. 2012, Wappler et al. 2012), and this is particularly true for the diverse paleofauna from Florissant.

**Figure 1.**
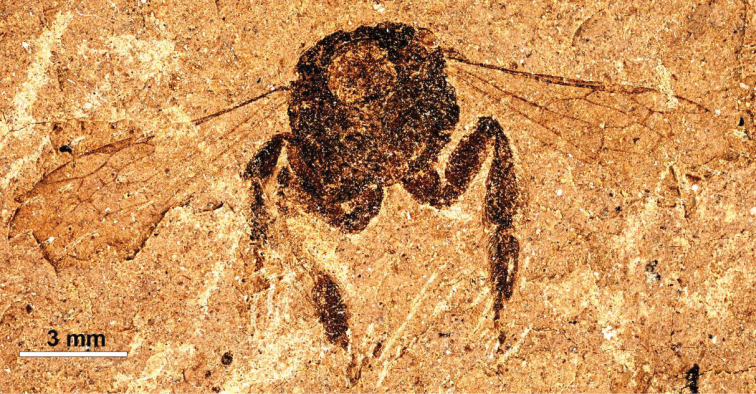
Photograph of holotype female of *Melitta willardi* Cockerell as preserved (UCM 18737). Specimen is preserved facing toward the viewer, with head missing (note the large opening representing the anterior thoracic fossa).

The highly fossiliferous shales of Florissant, Colorado have revealed 34 species and 19 genera belonging to several extant bee families: Apidae, Halictidae, Melittidae, Megachilidae, and Andrenidae (Michez et al. 2012). However, the material is often preserved with little or no relief and specimens typically have only the wing venation or limited structures of the legs and thorax discernible, making comparisons with extant clades difficult. Recently we have had the opportunity to re-examine the putative melittine bee from Florissant, *Melitta willardi* Cockerell 1909 (Figure 1), and to attempt a better understanding of its affinities with extant and other fossil taxa as determined by wing shape analyses.

## Material and methods

### Sampling

Given that *Melitta willardi* possesses three submarginal cells we sampled specimens from different extant subfamilies with the same arrangement of cells. All available subfamilies were included, with a maximum of 20 specimens per subfamily, and with a maximum of five specimens per species. We additionally sampled all species of *Melitta* available with a maximum of five specimens per species. The dataset also included eighteen extinct species [Apidae, Apinae: *Anthophorula persephone* Engel, 2012; *Bombus randeckensis* Wappler & Engel, 2012; *Electrapis meliponoïdes* (Buttel-Reepen, 1906); *Electrapis krishnorum* Engel, 2001; *Electrobombus samlandensis* Engel, 2001; *Eufriesea melissiflora* (Poinar, 1998); *Melikertes stilbonotus* (Engel, 2001); *Melissites trigona* Engel, 2001; *Paleohabropoda oudardi* Michez & Rasmont, 2009; *Protobombus basilaris* Engel, 2001; *Probombus hirsutus* Piton, 1940; *Succinapis goeleti* Engel, 2001; and *Thaumastobombus andreniformis* Engel, 2001; Halictidae, Halictinae: *Cyrtapis anomala* Cockerell, 1908; *Electrolictus antiquus* Engel, 2001; *Halictus petrefactus* Engel & Peñalver, 2006; *Ocymoromelitta sorella* Engel, 2002 and *Ocymoromelitta florissantella* (Cockerell, 1906)]. Since the holotype of *Melitta willardi* is a female, all individuals used in the morphometric analysis are females to avoid potential bias due to any sexual dimorphism. The assembled dataset comprised 360 specimens representing six families of Anthophila, 15 subfamilies, and 109 species (Table 1).

**Table 1.** Dataset for the geometric morphometric analysis including 360 specimens from 109 species and 15 subfamilies. N = number of specimens.

FAMILY	SUB-FAMILY	SPECIES	N
Andrenidae	Andreninae	*Andrena bicolor* Fabricius, 1775	5
		*Andrena boyerella* Dours, 1872	5
		*Andrena flavipes* Panzer, 1799	5
		*Andrena fulva* (Müller, 1766)	5
	Oxaeinae	*Oxaea flavescens* Klug, 1807	1
		*Oxaea fuscescens* Sichel, 1865	1
		*Oxaea* sp.	1
		*Protoxaea gloriosa* (Fox, 1893)	2
	Panurginae	*Borgatomelissa brevipennis* (Walker, 1871)	1
		*Melitturga clavicornis* (Latreille, 1808)	1
		*Melitturga taurica* Friese, 1922	5
		*Anthrenoïdes* sp.	2
		*Parapsaenythia puncticutis* (Vachal, 1909)	2
Apidae	Apinae	*Apis florea* Fabricius, 1787	5
		*Bombus mendax* Gerstäcker, 1869	5
		*Melissodes confusa* Cresson, 1878	5
		*Anthophora plumipes* (Pallas, 1772)	5
		*Paleohabropoda oudardi* Michez & Rasmont, 2009 †	1
		*Anthophorula persephone* Engel, 2012 †	1
		*Bombus randeckensis* Wappler & Engel, 2012 †	1
		*Electrapis krishnorum* Engel, 2001 †	1
		*Electrapis meliponoides* (Buttel-Reepen, 1906) †	1
		*Electrobombus samlandensis* Engel, 2001 †	1
		*Eufriesea melissiflora* (Poinar, 1998) †	1
		*Melikertes stilbonotus* (Engel, 2001) †	1
		*Melissites trigona* Engel, 2001 †	1
		*Protobombus basilaris* Engel, 2001 †	1
		*Probombus hirsutus* Piton, 1940 †	1
		*Succinapis goeleti* Engel, 2001 †	1
		*Thaumastobombus andreniformis* Engel, 2001 †	2
	Nomadinae	*Epeolus cruciger* (Panzer, 1799)	5
		*Nomada fabriciana* (Linnaeus, 1767)	5
		*Nomada flava* Panzer, 1798	5
		*Nomada goodeniana* (Kirby, 1802)	5
	Xylocopinae	*Ceratina chloris* (Illiger, 1806)	5
		*Ceratina dallatorreana* Friese, 1896	5
		*Xylocopa olivieri* (Lepeletier de Saint Fargeau, 1841)	5
		*Xylocopa violacea* (Linnaeus, 1758)	5
Colletidae	Colletinae	*Colletes cunicularius* (Linnaeus, 1761)	5
		*Colletes daviesanus* Smith, 1846	5
		*Colletes succinctus* (Linnaeus, 1758)	5
		*Leioproctus* sp.	5
	Diphaglossinae	*Cadeguala occidentalis* (Haliday, 1836)	1
		*Caupolicana gayi* Spinola, 1851	5
		*Caupolicana yarrowi* (Cresson, 1875)	3
		*Crawfordapis luctuosa* (Smith, 1861)	2
		*Diphaglossa gayi* Spinola, 1851	3
		*Mydrosoma bohartorum* Michener, 1986	1
		*Ptiloglossa guinnae* Roberts, 1971	1
		*Ptiloglossa pretiosa* (Friese, 1898)	4
Halictidae	Halictinae	*Augochlorella striata* (Packer, 1990)	5
		*Halictus ligatus* Say, 1837	5
		*Ruizantheda nigrocaerulea* (Spinola, 1871)	5
		*Thrinchostoma kandti* Blüthgen, 1930	5
		*Cyrtapis anomala* Cockerell 1908 †	1
		*Electrolictus antiquus* Engel 2001 †	1
		*Halictus petrefactus* Engel & Peñalver 2006 †	1
		*Ocymoromelitta florissantella* Cockerell 1906 †	1
		*Ocymoromelitta sorella* Engel, 2002 †	1
	Nomiinae	*Dieunomia nevadensis* (Cresson, 1874)	1
		*Halictonomia decemmaculata* (Friese, 1900)	2
		*Lipotriches australica* (Smith, 1875)	1
		*Lipotriches modesta* (Smith, 1862)	5
		*Nomia melanderi* Cockerell, 1906	1
		*Nomia diversipes* Latreille, 1806	5
		*Pseudapis diversipes* (Latreille, 1806)	5
	Nomioidinae	*Ceylalictus variegatus* (Olivier, 1789)	5
		*Nomioides facilis* (Rossi, 1853)	5
		*Nomioides minutissimus* (Rossi, 1790)	1
	Rophitinae	*Systropha curvicornis* (Scopoli, 1770)	2
		*Systropha maroccana* Warncke, 1977	3
		*Systropha pici* Pérez, 1895	2
		*Systropha planidens* Giraud, 1861	5
		*Systropha* sp.	5
Megachilidae	Fideliinae	*Fidelia kobrowi* Brauns, 1905	5
		*Fidelia paradoxa* Friese, 1899	5
		*Fidelia villosa* Brauns, 1902	1
		*Fideliopsis major* (Friese, 1911)	2
Melittidae	Meganomiinae	*Meganomia andersoni* (Meade-Waldo, 1916)	2
		*Meganomia binghami* (Cockerell, 1909)	5
	Melittinae	*Rediviva intermixta* (Cockerell, 1934)	5
		*Rediviva longimanus* Michener, 1981	3
		*Melitta americana* Smith, 1853	3
		*Melitta arrogans* Smith, 1879	5
		*Melitta bicollaris* Warncke, 1973	5
		*Melitta californica* Viereck, 1909	1
		*Melitta cameroni* (Cockerell, 1910)	5
		*Melitta dimidiata* Morawitz, 1876	5
		*Melitta eickworti* Snelling & Stage, 1995	3
		*Melitta ezoana* Yasumatsu & Hirashima, 1956	5
		*Melitta haemorrhoidalis* (Fabricius, 1775)	5
		*Melitta hispanica* Friese, 1900	5
		*Melitta harrietae* (Bingham, 1897)	5
		*Melitta japonica* Yasumatsu & Hirashima, 1956	4
		*Melitta magnifica* Michez, 2012	3
		*Melitta melittoides* (Viereck, 1909)	2
		*Melitta melanura* (Nylander, 1852)	5
		*Melitta murciana* Warncke, 1973	5
		*Melitta seitzi* Alfken, 1927	1
		*Melitta schultzei* Friese, 1909	1
		*Melitta sibirica* (Morawitz, 1888)	5
		*Melitta aegyptiaca* (Radoszkowski, 1891)	5
		*Melitta leporina* (Panzer, 1799)	5
		*Melitta maura* (Pérez, 1896)	5
		*Melitta nigricans* Alfken, 1905	5
		*Melitta schmiedeknechti* Friese, 1898	5
		*Melitta tricincta* Kirby, 1802	5
		*Melitta avontuurensis* Michez & Kuhlmann, 2014	1
		*Melitta richtersveldensis* Michez & Kuhlmann, 2014	5
**Total = 360**			

### Morphometric and statistical analyses

Taxonomic affinities of the fossil were evaluated based on wing shape. Wing venation is used widely in insect taxonomy and can provide many informative features for phylogenetic analyses and for many Late Paleozoic taxa is sometimes the only form of available data (e.g. Gumiel et al. 2003, Pretorius 2005). Moreover, use of the wings has significant advantages compared to other organs, i.e., they are relatively rigid, articulated, 2D structures that present a large number of useful landmarks formed by the homologous intersections of veins. Geometric morphometrics is a procedure which aims at quantifying and analyzing the overall shape of a structure (Bookstein 1991, Rohlf and Marcus 1993, Adams et al. 2004), and can provide a powerful tool in paleontology for discriminating taxa at different levels as well as for discussing taxonomic affinities between extinct and extant taxa (Roberts 2008, Michez et al. 2009, De Meulemeester et al. 2012, Wappler et al. 2012). The holotype of *Melitta willardi* does not exhibit any signs of post-mortem tectonic deformation, meaning that the venation observed is reflective of as it was in life and did not require any compensation to adjust for taphonomic or diagenetic alteration.

**Figure 2.**
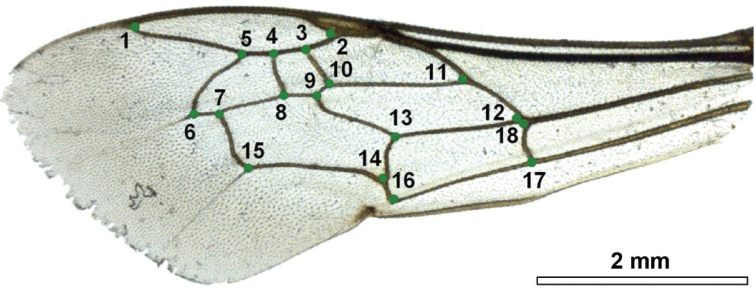
Right forewing of a female of *Melitta leporina* (Panzer) with the 18 landmarks indicated to describe the shape.

The right forewings of 360 female specimens were initially photographed using an Olympus SZ010 binocular coupled with a Nikon D70 camera. Photographs were gathered in one TPS file using tps-UTIL 1.56 (Rohlf 2013a). To capture the shape, two dimensional Cartesian coordinates of 18 landmarks (Figure 2) were digitized by tps-DIG 2.17 (Rohlf 2013b). Both right and symmetrized-left wings of *Melitta willardi* were digitized by four experimenters (AD, MD, TD, DM) to obtain an objective and robust identification. All landmark configurations were scaled, translated, and rotated against the consensus configuration by the generalized least square Procrustes superimposition method (Bookstein 1991). The superimposition was performed using R functions of the package “geomorph” (Adams and Otárola-Castillo 2013). The aligned landmark configurations were projected into the Euclidean space tangent to the curved Kendall’s shape space to aid further statistical analyses. The closeness of the tangent space to the curved shape space was tested by calculating the least-squares regression slope and the correlation coefficient between the Procrustes distances in the shape space with the Euclidean distances in the tangent space (Rohlf 1999). This variation amplitude of our dataset was calculated with tps-SMALL 1.25 (Rohlf 2013c).

Prior to the assignment of the fossil, shape variation within the reference dataset and discrimination of the different taxa was assessed by Linear Discriminant Analyses (LDA) of the projected aligned configurations of landmarks, with subfamily levels as *a priori* grouping by using the software R version 3.0.2 (2013, http://www.R-project.org/). The effectiveness of the LDA for discriminating subfamilies was assessed by the percentages of individuals correctly classified to their original taxon (hit-ratio, HR) in a leave-one-out cross-validation procedure based on the posterior probabilities of assignment. Given the observed scores of an “unknown”, the posterior probability (PP) equals the probability of the unit to belong to one group compared to all others. The unit is consequently assigned to the group for which the posterior probability is the highest (Huberty and Olejnik 2006).

Taxonomic affinities of the fossil were assessed based on their score in the predictive discriminant space of shapes. After superimposition of the 368 landmark configurations (i.e. corresponding to the reference dataset and the fossil), aligned coordinates of the 360 specimens from the reference dataset were used to calculate the LDA. We included *a posteriori* the eight aligned landmark configurations of *Melitta willardi* in the computed LDA space as “unknown” specimens and calculated their score. Assignments of the fossil configurations were estimated by calculating the Mahalanobis Distance (MD) between “unknowns” and group mean of each subfamily. We also calculated posterior probabilities of assignment to confirm the assignment to one taxon.

In order to assess the taxonomic affinities of *Melitta willardi* with the family Andrenidae, PCA was computed to visualize shape affinities between the fossil and andrenid subfamilies.

## Results

### Morphometric analysis

The regression coefficient between the Procrustes distances and the Euclidean distances is close to 1 (0.9999). This means that the linear tangent space closely approximates the shape space, thereby permitting us to be confident in the variation amplitude of our dataset.

In LDA space with subfamily *a priori* grouping, discrimination of the 15 groups are effective, with a cross-validated HR of 98.61% (e.g., 5 misclassified specimens), and 10 of the 15 subfamilies that account for a HR of 100% (Table 2). Other subfamilies have a HR between 90% and 99%. Due to sampling size within groups, the HR drastically drop down when a single specimen is misclassified. This is the case for the five groups with HR lower than 100%. Cross-validation assignment (Table 2) allows us to be confident in the group discrimination at subfamily level.

**Table 2.** Cross-validation assignment in LDA space with subfamily *a priori* grouping (original groups are along the rows, predicted groups are along the columns). HR = Hit ratio.

	Andreninae	Apinae	Colletinae	Diphaglossinae	Fideliinae	Halictinae	Meganomiinae	Melittinae	Nomadinae	Nomiinae	Nomioidinae	Oxaeinae	Panurginae	Rophitinae	Xylocopinae	HR (%)
Andreninae	20	-	-	-	-	-	-	-	-	-	-	-	-	-	-	100
Apinae	-	33	-	1	-	-	-	-	-	-	-	-	-	-	-	97
Colletinae	-	-	20	-	-	-	-	-	-	-	-	-	-	-	-	100
Diphaglossinae	-	1	-	19	-	-	-	-	-	-	-	-	-	-	-	95
Fideliinae	-	-	-	-	13	-	-	-	-	-	-	-	-	-	-	100
Halictinae	-	1	-	-	-	24	-	-	-	-	-	-	-	-	-	96
Meganomiinae	-	-	-	-	-	-	7	-	-	-	-	-	-	-	-	100
Melittinae	-	-	-	-	-	-	-	117	-	-	-	-	-	-	-	100
Nomadinae	-	-	-	-	-	-	-	-	20	-	-	-	-	-	-	100
Nomiinae	-	-	-	-	-	-	-	-	-	20	-	-	-	-	-	100
Nomioidinae	-	-	-	-	-	-	-	-	-	-	11	-	-	-	-	100
Oxaeinae	-	-	-	-	-	-	-	-	-	-	-	5	-	-	-	100
Panurginae	1	-	-	-	-	-	-	-	-	-	-	-	10	-	-	91
Rophitinae	-	-	-	-	-	1	-	-	-	-	-	-	-	16	-	94
Xylocopinae	-	-	-	-	-	-	-	-	-	-	-	-	-	-	20	100

**Figure 3.**
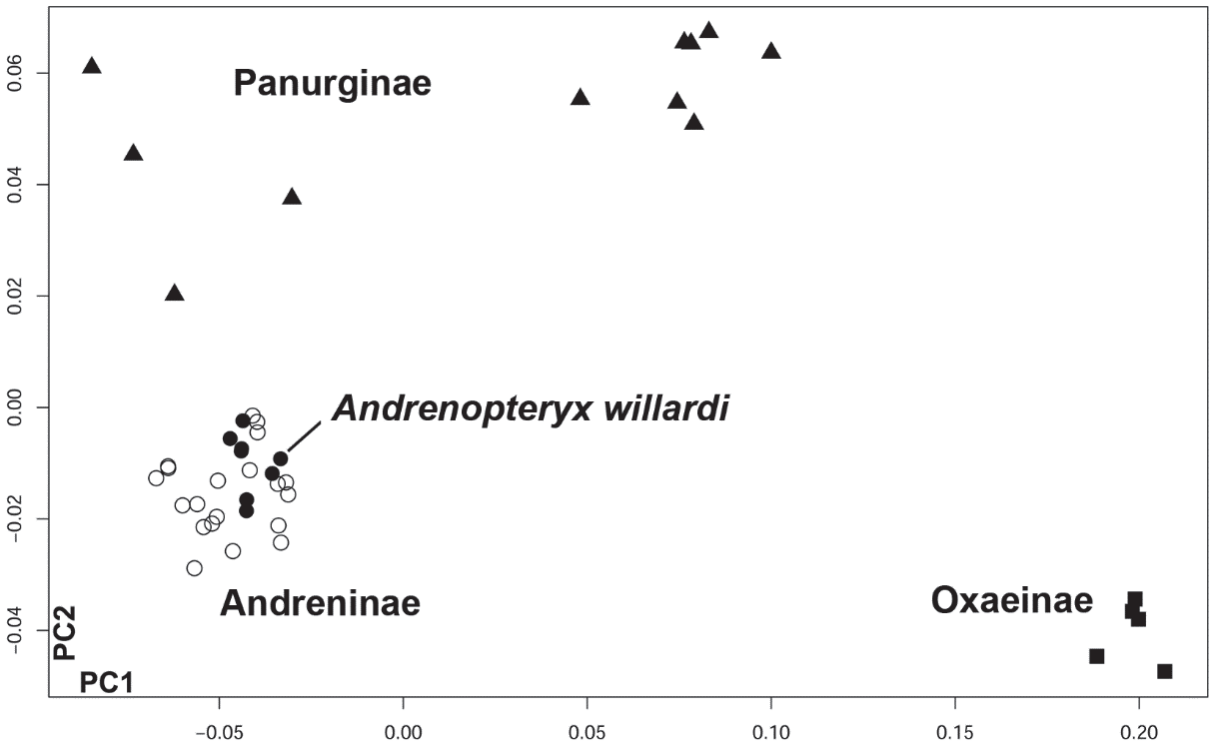
Distribution of extant examined andrenid (36 specimens) and the eight landmark configurations of *Andrenopteryx willardi* (*), along the first two PC axis (PC1 = 72%, PC2 = 11%).

All of the 109 specimens of *Melitta* were correctly classified to their original taxon (Melittinae) in the leave-one-out cross-validation procedure. However, the eight landmark configurations of *Melitta willardi* are assigned to Andreninae (MDs = 2.72 – 4.84; PPs = 0.9999 – 1). Taxonomic affinities of the fossil were also assessed based on non-supervised analyses within Andrenidae. In the morphometric space defined by the PCA, the fossil is undoubtedly clustered with the subfamily Andreninae (Figure 3).

## Systematic palaeontology

### Family: Andrenidae Latreille, 1802
Subfamily: Andreninae Latreille, 1802

#### Genus Andrenopteryx

Dewulf & Engel gen. n.

http://zoobank.org/2A2AF004-6EEB-47DE-B13F-B91378CF3557

http://species-id.net/wiki/Andrenopteryx

##### Type species.

*Melitta willardi* Cockerell, 1909.

##### Included species.

The genus presently includes only the type species, *Andrenopteryx willardi* (Cockerel, 1909), comb. n.

##### Diagnosis.

♀: Forewing with three submarginal cells, first submarginal cell largest, second smallest; r-rs long, about as long as anterior border of second submarginal cell; anterior border of second submarginal cell not dramatically shorter than that of third submarginal cell; 1rs-m relatively straight; 2rs-m greatly arched apical in posterior half; 1m-cu entering second submarginal cell near midpoint; 2m-cu entering third marginal cell at apical third of cell length, 2m-cu relatively straight; pterostigma linear, much longer than wide, border inside marginal cell relatively straight; marginal cell with acutely rounded apex, not truncate or appendiculate, apex on costal margin, apical most abscissa Rs relatively straight such that marginal cell apex tapers gradually in width from 2rs-m to apex. Pilosity well developed; flocculus absent; scopa present on metafemur and metabasitarsus; metabasitarsus more than half as long as metatibia; pretarsal claws with minute inner tooth. ♂: Unknown.

##### Etymology.

The new genus-group name is a combination of *Andrena*, type genus of the subfamily Andreninae, and -*pteryx*, meaning “wing”. The name is feminine and refers to the “*Andrena*-like” venation of the wings.

## Discussion

### Position of *Andrenopteryx* gen. n. in Anthophila

The wings of *Andrenopteryx* gen. n. have three submarginal cells, suggesting that the genus does not probably belong to subfamilies such as Xeromelissinae, Hylaeinae, Euryglossinae (all Colletidae), Dasypodainae (Melittidae), Megachilinae (Megachilidae), or various tribes among the Apidae (i.e., Allodapini, Ammobatini, Ammobatoidini, Biastini, Boreallodapini, Caenoprosopidini, Ctenoplectrini, Neolarrini, and Townsendiellini). Furthermore, *Andrenopteryx* gen. n. clearly possesses pollen-collecting structures, suggesting that the fossil was probably not cleptoparasitic and accordingly those genera may also be excluded (cleptoparasitic genera occur in various families, see Michener 2007). The GM analysis of the wing shape of *Andrenopteryx* gen. n. suggests that this fossil belongs to the Andrenidae (see previous results, vide supra). Nevertheless, diagnostic features of Andrenidae such as the two subantennal sulci and the short to long pointed glossa are not preserved in the only available specimen of the species.

Assuming that its clustering among Andrenidae is an accurate reflection of its relationships, among andrenids the three submarginals cells excludes placement among most of the Panurginae. The species has a long marginal cell with an acutely curved apex that lies along the costal margin as in Andreninae, while the other subfamilies have a marginal cell with a truncate apex (Michener 2007). The holotype clearly possesses a scopa which is limited to the metafemur and metabasitarsus and without a flocculus, unlike the diverse extant genus *Andrena* Fabricius. The metabasitarsus is more than half as long as the metatibia, in stark contrast to the form present in *Megandrena* Cockerell. Lastly, *Andrenopteryx* gen. n. does not have an enlarged inner tooth on the pretarsal claws, and therefore is distinct from the southern South American genus *Orphana* Vachal. Thus, while Cockerell’s species certainly is best placed in the Andreninae it seems generically distinct and this has served as the basis for our decision to describe a new genus.

### Geometric morphometrics of wing shape and *Andrenopteryx willardi*

Wing shape analyses were successfully employed in previous studies to discriminate extant bee taxa at various classificatory levels, from subspecies to tribes (e.g., Kandemir et al. 2011, De Meulemeester et al. 2012). In addition, these analyses are sufficient to confidently associate bee fossils with extant groups (e.g., Michez et al. 2009, De Meulemeester et al. 2012, Wappler et al. 2012), and this lends increased confidence to the affinities of *Andrenopteryx willardi* as outlined above.

Cockerell (1909) mentioned some features that for him indicated that his fossil species was referable to *Melitta*. He noted the three submarginal cells, the particular form of the pterostigma, the scopa confined to the metafemur and metabasitarsus, and the absence of a flocculus. However, a majority of these similarities are unfounded and not indicative of *Melitta*, and in fact some are more suggestive of Andreninae. First, the proportions of the submarginal cells are more similar to Andrenidae than any melittid. Second, the second submarginal cell does not receive the 1m-cu well before its midpoint, and this is true for both the left and right forewings. Thus, even based on the evidence available to Cockerell and from his description the fossil should not be placed within *Melitta*, and it is peculiar to us why he made such a taxonomic decision.

Based on the discovery that Cockerell’s fossil *Melitta* is more likely an andrenine, some previous hypotheses regarding the biogeography of North American bees require reconsideration. Michez and Eardley (2007) speculated the presence of *Melitta* in North America during the Oligocene based on Cockerell’s (1909) assertion of the taxonomic identity of *Andrenopteryx willardi*, and Dellicour et al. (2014) demonstrated that North American species of *Melitta* form a derived clade within the genus. There is now no evidence for Michez and Eardley’s scenario. The origin of *Melitta* could be more recent than previously hypothesized and Dellicour et al.’s North American clade could have entered and diversified on the continent during the Neogene. In contrast, the record of Andreninae in North America during the Oligocene is now corroborated by the present fossil. There are additional records of putative andrenines from Florissant, such as *Lithandrena saxorum* Cockerell, 1906, *Pelandrena reducta* Cockerell 1909, and five additional species Cockerell placed in *Andrena* (Michez et al. 2012), but these are in need of re-evaluation. It is hoped that these species may also be subjected to morphometric analyses and their relationships clarified.

### The importance of the Florissant shales

The bees of the Florissant shale have been ignored for a long time (Engel 2002). It was Cockerell’s intention to document the whole fossil fauna and flora from Colorado and this partly drove his efforts to document the known bee remains from these deposits (Cockerell 1927, Engel 2002). Cockerell, who largely relied on a hand lens to study specimens, often based his hypotheses regarding the placement of particular fossils on the their general habitus, or relied on a suite of traits recognized nowadays as not indicative of those same families, subfamilies, and even genera. For example, many of the traits concerning wing shape such as the relative positions of the rs-m or m-cu crossveins are quite variable within individual families. Subsequent to Cockerell, Zeuner and Manning (1976) tried to evaluate the Florissant fossil bees, but they based their work solely from Cockerell’s original descriptions and did not examine type material. Zeuner and Manning’s monograph is further compromised by the fact that both authors died before the work was completed, leaving behind only notes that were subsequently cobbled together to form the publication, and this explains its poor quality and limited utility (Engel 2002). The only other works were brief accounts by Engel (2001b, 2002), who attempted to re-evaluate Cockerell’s Florissant halictines as well as newly discovered material, and to describe a new large carpenter bee. Outside of this, the Florissant fossil bee fauna has remained dormant and given that it is one of the most diverse and specimen-rich deposits for the Anthophila, it is all the more imperative that its species be properly evaluated in a modern context. We hope that this brief treatment of one such species will inspire more investigations into the fossil bees from Colorado.

## Supplementary Material

XML Treatment for Andrenopteryx
